# Mental health outcomes of offspring exposed to parental intimate partner violence in Rwanda

**DOI:** 10.1186/s12887-024-04884-y

**Published:** 2024-07-01

**Authors:** Claire Bahati, Amina Nyiranteziryayo, Josias Izabayo, Pauline Atete, Vincent Sezibera, Leon Mutesa

**Affiliations:** 1 Department of Clinical Psychology, College of Medicine and Health Sciences, University of Rwanda, Kigali, Rwanda; 2 Centre for Mental Health, College of Medicine and Health Sciences, University of Rwanda, Kigali, Rwanda; 3 Centre for Human Genetics, College of Medicine and Health Sciences, University of Rwanda, Kigali, Rwanda

**Keywords:** Intimate partner violence, Mental health outcomes, Offspring, Rwanda

## Abstract

**Background:**

Children who witness parental intimate partner violence (IPV) are more likely to develop mental health issues compared to those who do not witness such violence.

**Objective:**

The main objective of this study is to assess the association between parental intimate partner violence and child mental health outcomes.

**Methodology:**

This cross-sectional study involved 548 participants divided into two groups: parents (*N* = 304) and offspring (*N* = 244). The participants were recruited from Mageragere Sector in the City of Kigali (urban), as well as Mbazi and Ruhashya sectors in Huye District (rural). To assess the difference about mental difficulties reported by the offspring, a Mann-Whitney U test was employed to compare the responses of parents and their children on mental health outcomes. Additionally, multiple linear regression analysis was conducted to explore the association between parental intimate partner violence (IPV) and the mental health outcomes of their offspring.

**Results:**

The results highlighted significant levels of mental and emotional challenges in children, as reported by both parents and the children themselves. Depression and youth conduct problems were more prevalent among the children compared to their parents, whereas anxiety and irritability were more commonly reported by parents than by their children. Intimate partner violence showed to be a predictor of irritability and anxiety symptoms in offspring. In terms of irritability, depression, and youth conduct problems they were identified as predictors of anxiety symptoms. Particularly, anxiety and irritability were revealed to predict youth conduct problems.

**Conclusion:**

The study indicates that parental intimate partner violence (IPV) has an impact on the mental well-being of their offspring. Furthermore, it was observed that there is not only a correlation between IPV and poor mental health outcomes, but also a connection between different mental conditions, implying that children exposed to IPV are more prone to experiencing a range of mental issues. As a result, intervention programs should place emphasis on addressing the mental disorders of both parents and children.

## Background

Domestic violence (DV) is reported globally as a major challenge for development in general, and particularly, for the cohesion of communities and families [1]. Intimate partner violence (IPV) is one of the forms of domestic violence as it opposes two partners in an intimacy relationship. IPV defined as physical violence, sexual violence, stalking and psychological aggression (including coercive tactics) by a current or former intimate partner (i.e., spouse, boyfriend/girlfriend, dating partner, or ongoing sexual partner) [2], is associated with adverse health outcomes including poor physical health [3], maxillofacial injuries [4, 5], mental difficulties [3, 6–12]; poor reproductive health [13, 14], and intimate partner homicide [15].

Increase in DV and IPV is a concern for Rwanda, as it is worldwide. Rwanda Demographic Health Surveys (RDHS) have shown that the prevalence of women who have been victims of spousal violence (physical, sexual, psychological and socio-economic) has increased from 40% in 2015 to 46% in 2020 [16], placing Rwanda among the countries with the high rates of domestic and marital violence. Rurangirwa and colleagues found evidence of association between experience of IPV during pregnancy and mental disorders among women such as major depression, suicide ideations, generalized anxiety, and posttraumatic stress disorders (PTSD) [17]. Recently scholars in Rwanda found increased odds of mental disorders in participants with a history of IPV exposure, as compared to non-exposed ones [18]. In Rwanda, however, little to nothing is known about the effects of exposure to IPV to mental health of the children.

Existing evidence on the relationship between the exposure to IPV and mental problems among children is mostly generated from high income countries (HIC), whilst a few studies conducted in poor resources settings also found evidence of association between the exposure to IPV and increased risk of developing mental disorders among children. In Uganda Devries et al. found that the odds of having mental difficulties were four times higher for students who had witnessed and experienced violence compared to those who did not [11], while Chander and colleagues found the association between caregiver experience of IPV and child behavior difficulties [7]. Moreover, understanding the impact of IPV on mental health necessitates consideration of the sociocultural, economic, political-legal, and historical factors that shape victims’ actions and responses to violence [19]. Gender and power norms play a significant role in perpetuating IPV and influencing survivors’ access to support services and resources [20]. By contextualizing IPV within these broader socio-cultural frameworks, we can develop more effective interventions to address mental health outcomes in Rwanda.

Common reported mental difficulties in children who are exposed to IPV include externalizing behaviors such as aggression and delinquency, and internalizing behavior such as anxiety, depression, and withdraw. Moylan and colleagues found that child exposure to violence was associated with delinquency, depression, and withdraw [9], while Chander et al. found a significant relationship between IPV and child internalizing behavior [7]. Additionally, being raised by parents who experience IPV can lead to the internalization of trauma among children which is transferred through parents to children’s interactions. This intergenerational transmission of trauma has been linked to various mental health issues, including anxiety, depression, and PTSD [21]. By exploring this phenomenon in Rwanda, we aim to provide a deeper understanding of the complex dynamics between IPV exposure and mental health outcomes of the children. It is worth noting that women often refrain from disclosing the violence they endure for various reasons, including fear of reprisal, societal stigma, and cultural norms. Consequently, these issues may remain concealed until a critical incident of extreme violence occurs. This delayed disclosure further exacerbates the psychological impact on both the women experiencing IPV and their children [19]. Associations between exposure to IPV and high scores (meaning mental difficulties) on strengths and difficulties questionnaire (SDQ) were also reported [10, 11].

In Rwanda, a handful of publications analyzing the relationship between IPV, and mental health outcomes are available [17, 18, 22, 23], and despite being a public health issue, there is a huge knowledge gap about the effect of IPV on mental health of the children exposed to parental IPV. Studies have shown that children raised in households where IPV occurs are at increased risk of experiencing psychological distress and exhibiting aggressive behavior themselves [24]. The cycle of violence perpetuates as children internalize and mimic the aggressive behaviors witnessed at home [25]. The present study seeks to fill the existing gap by investigating the effects of parental IPV on children mental health outcomes.

## Methods

### Setting

The participants for the study were recruited from three sectors namely Mageragere in the City of Kigali, and Mbazi and Ruhashya sectors in Huye District. These three sectors were selected because of the existence of local initiatives that address the issue of IPV, particularly the Life Wounds Healing Association (LIWOHA). LIWOHA is a local non-governmental organization which addresses mental difficulties using community approach through psychoeducation and coaching. Prior to being recruited by LIWOHA for a 13-day workshops on raising awareness about DV (3 days), healing individual wounds caused by DV (5 days), and conflict management and mediation (5 days); all adults participants to the study had been identified by the local government authorities as couples that had had ongoing disputes in their families.

### Sampling procedures

A purposive sampling procedure was followed during the recruitment of the participants to the study. Parents were recruited if: (1) were legally married, (2) had at least one 7–17 years-old child, (3) had been identified by the local government authorities as couples that had had disputes, (4) were willing to participate in the study without a compensation. Children were recruited into the study if: (1) their parents were recruited into the study, (2) they were between 7 and 17 years old, and (3) obtained the parents’ assent to participate in the study. A total of 548 participants were recruited in this study including 304 parents and 244 offspring.

### Measures

All measures were developed and adapted for the Rwandan context [26]. All the questionnaires were translated into Kinyarwanda by five bilingual psychologists fluent in both English and Kinyarwanda. The back-translation was performed by two other psychologists proficient in both languages. The back-translated version was then compared to the original, and any discrepancies were discussed and resolved.

### The HITS (hurt, insult, threaten, screaming)

The HITS instrument was used to measure intimate partner violence [27]. The HITS is a seven items scale with a 4-point scale in the form: never (0 point), sometimes (1 point), fairly often (2 points) and frequently (3 points). The HITS was translated from English to Kinyarwanda and the translated version demonstrated very good internal consistency and validity indices (Cronbach’s alpha = 0.83). The strengths of the HITS instrument lie on its brevity as well as its capacity to capture both physical and non-physical (verbal, psychological, overcontrol behavior) form of violence.

### Youth conduct problems scale-rwanda (YCPS-R)

The YCPS-R is an 11 items assessment scale for conduct problems among youth developed for use in Rwanda beta [26]. The scale was validated in previous study in Rwanda [28], and has demonstrated a good reliability (α = 0.87) in the present study. Compared to other instruments (MINI for children, WHO-Disability Assessment Schedule Child Version) YCPS-R demonstrated the ability to discriminate between youth with and without conduct disorder. Most importantly, the YCPS-R was developed from a locally derived measures on Rwandan youth with conduct problems. Specific clinical cut off values were applied to different categories of participants; children (5), mother parents (14), and father parents (9) to determine the level of clinically diagnosable conduct problems.

### The adapted youth self-report (YSR)-internalizing subscale

The adapted YSR is 23-item scale that measures anxiety, depression, and withdrawal symptoms and has demonstrated good reliability and validity among Rwandan youth [26]. The YSR used in this study has demonstrated good internal consistency and validity (α = 0.92). A clinical cut-off value of 67 for T-scores was used to determine the diagnosable cases for emotional functioning.

### The center for epidemiological studies depression scale for children (CES-DC)

The CES-DC, a 4-point Likert scale scoring from 0 (never) to 3 (often), was used to assess depression symptoms, the adapted version [26,29], included local wording to capture local depression-like problems *agahinda kenshi* (persistent sorrow) and *kwiheba* (severe hopelessness). The adapted CES-DC Kinyarwanda version (30 items) demonstrated excellent psychometric properties (Cronbach’s alpha = 0.91) and 30 cut-off point was used to determine clinical diagnosable depression [19, 22].

### The adapted irritability questionnaire (IRQ)

The IRQ (27-item scale) is an adapted version from the 21-item Irritability Questionnaire to fit Rwandan context by adding new items generated from qualitative data [26, 30].

### Statistical analysis

Sum scores were computed for different scales used for the present study including HITS, YCPS-R, YSR, IRQ and CES-DC. After computing sum scores, dichotomous variables were created using the cut off values found in the literature to determine participants who met criteria for possible clinical cases. For YRS scale, transformation of raw values into T-scores was performed to be able to determine a cut off level for emotional impairment. Frequency tables were used to describe the social and demographic characteristics of the sample and as well as mental difficulties for the children. In addition, correlation analysis was conducted to identify the level of associations existing between different mental difficulties among the offspring. Mann-Whitney U test, was conducted to understand whether the median differences between parents reports and offspring reports about mental difficulties among offspring were statistically significant, furthermore, the multiple linear regression was used to examine the association between intimate partner violence and offspring’s menta health outcomes. All the statistical analysis were performed using SPSS version 25.

## Results

Table 1 provides a description about characteristics of the children and parents interviewed during study. In total, 548 participants were interviewed including 244 children and 304 parents. The mean age for children was 12.2(SD = 2.6) with the minimum and maximum age of seven and 17 respectively. Out of 244 children who were interviewed 52.5% were girls and 47.5% were boys. Most of the children (88.9%) were in the primary school, 9.4% were in the secondary school while 1.6% were illiterate. At the time of the interview, 88.3% (*n* = 212) of the children reported being exposed to IPV while 93.3% believed that IPV happening within their families had effect on them. Most of the children had been exposed to IPV for the period between 1 and 5 years and less than 5% of them had been exposed to IPV for a period of more than 10 years. In total 304 parents were interviewed with female participants representing 68.1% of the sample. The mean age of the parents was 44 years (SD = 9.7) with the minimum and maximum age of 27 and 76 years respectively. Also, more than three quarters of the parents had primary school education level, 13.8% were illiterate, and 0.3% had attended university education. As far as IPV is concerned, 86.4% of the parents were exposed to current IPV and 94.4% believed that their children were affected by IPV. About 40% of the parents had experienced IPV for the period between 1 and 5 years whilst 30% of them had experienced violence for the period between 11 and 40 years.

**Table 1:** Demographic and IPV characteristics of the children and parents

		Children		Parents	
		Frequency	Percentage	Frequency	Percentage
***Sex***	Female	128	52.5	207	68.1
	Male	116	47.5	97	31.9
***Age groups***	7–10	72	29.5	-	-
	11–14	113	46.3	-	-
	15–17	59	24.2	-	-
	27–36	-	-	77	25.3
	37–46	-	-	108	35.5
	47–56	-	-	84	27.6
	57–66	-	-	29	9.5
	67–76	-	-	6	2
***Level of education***	Illiterate	4	1.6	42	13.8
	Primary	217	88.9	246	80.9
	Secondary	23	9.4	12	3.9
	University			1	0.3
	Vocational			3	1
***Current IPV Exposure***	Yes	212	88.3	260	86.4
	No	28	11.7	41	13.6
***Effect of IPV on children***	Yes	224	93.3	284	94.4
	No	16	6.7	17	5.6
***Duration of IPV Exposure***	1–5	157	69.8	119	40.5
	6–10	60	26.7	87	29.6
	11–40	8	3.6	88	29.9

Table 2 reports on the mental and emotional difficulties in the children exposed to IPV as reported by both the parents and children themselves. Irritability was the most emotional difficulty that children had, and that is according to both parents and children. Parents perceived that 40% of the children were irritable while children believed that 33.9% of them were irritable. According to children account 26.25% were depressed and 28.5% had developed conduct problems. Also, using the YSR scale children reported that 4.2% had developed internalizing and externalizing behaviors which tend towards anxiety, depression, and feeling over controlled. According to parents reports 23.3% of the children were depressed and that 8.3% of the children had developed emotional and behavioral problems. Female parents rated the development of conduct problems of the children higher (19.8%) compared with male parents (9.6%).

**Table 2:** Mental and emotional difficulties among offspring exposed to parental IPV

		*N*	%	SE	95% CI
Emotional/Behavior problems (YSR)	Parent report	304	8.3	1.6	5.6–11.8
	Child report	244	4.18	1.30	2.17–7.29
Irritability (IRQ)	Parent report	304	40.1	2.8	34.6–45.7
	Child report	244	33.88	3.04	28.14-40
Depression (CES-DC)	Parent report	304	23.3	2.4	18.8–28.3
	Child report	244	26.25	2.84	20.99–32.1
Conduct problems (YCPS)	Mother report	207	19.8	2.3	15.6–24.6
	Father report	97	9.6	1.7	6.6–13.3
	Child report	244	28.51	2.90	23.1-34.43

YCPS-R, Youth Conduct Problems Scale-Rwanda

YSR, Adapted Youth Self-Report -Internalizing Subscale

CES-DC, The Center for Epidemiological Studies Depression Scale for Children

The HITS, Hurt, Insult, Threaten, Screaming

IRQ, The Adapted Irritability Questionnaire

Table 3 presents information regarding the correlations between Intimate Partner Violence (IPV) and internalizing and externalizing emotional and behavioral problems among the children. The results revealed significant positive correlations between IPV and child mental health outcomes, including depression, anxiety, irritability, and youth conduct problems (*p* < 0.01). These findings indicate that higher levels of IPV exposure are associated with increased likelihood of experiencing these mental health difficulties. Furthermore, the results also demonstrated significant intercorrelations among the mental health outcomes themselves (*p* < 0.01), suggesting that these difficulties are interconnected and tend to co-occur among children affected by IPV.

**Table 3:** Summary of correlations, Means and Standard Deviations for Scores on the HITS, CES-DC, YSR, IRQ, YCPS

Measure	HITS	CES-DC	YSR	IRQ	YCPS
HITS	—				
CES-DC	0.455^**^	--			
YSR	0.512^**^	0.812^**^	--		
IRQ	0.469^**^	0.774^**^	0.774^**^	--	
YCPS	0.298^**^	0.469^**^	0.490^**^	0.493^**^	--
M	9	22.6	12.68	26.17	4.38
SD	5.33	15.17	10.51	13.63	5.75

YCPS-R, Youth Conduct Problems Scale-Rwanda

YSR, Adapted Youth Self-Report -Internalizing Subscale

CES-DC, The Center for Epidemiological Studies Depression Scale for Children

The HITS, Hurt, Insult, Threaten, Screaming

IRQ, The Adapted Irritability Questionnaire

**p < 0.01

To evaluate the difference in perceptions of children’s mental health between parents and their children, a Mann-Whitney U test was utilized. The test results indicated that there was no statistically significant difference in the perceptions of parents and children regarding children’s mental health (*p* > 0.05) (see Table 4).

**Table 4:** Comparisons of mean scores on CES-DC, YSR, IRQ, YCPS-R

	Children’s report	Parent’s report	*U*	*P* value
Measure	Median	Mean		
Depression (CES-DC)	20.0	18.0	33696.5	0.134
Anxiety (YSR)	10.0	10.0	33680.5	0.102
Irritability (IRQ)	24.0	24.0	36088.5	0.803
Youth Conduct Problem (YCPS)	2.0	3.0	34570.0	0.245

YCPS-R, Youth Conduct Problems Scale-Rwanda

YSR, Adapted Youth Self-Report -Internalizing Subscale

CES-DC, The Center for Epidemiological Studies Depression Scale for Children

The HITS, Hurt, Insult, Threaten, Screaming

IRQ, The Adapted Irritability Questionnaire

The model comprised of parental intimate partner violence (IPV) and other symptoms of child mental health outcomes (depression, irritability, and youth conduct problems) explained 73% of the total variance of anxiety symptoms. IPV was significantly associated with anxiety (β = 0.133, *p* < 0.001). Additionally, the other mental health outcomes included in the model, such as irritability (β = 0.298, *p* < 0.001), youth conduct problems (β = 0.076, *p* < 0.004), and depression (β = 0.485, *p* < 0.001), were significantly associated with anxiety among children (Table 5).

**Table 5:** The association between parental intimate partner violence and child’s anxiety Symptoms

Variables	Unstandardized coefficients		Standardized coefficients	t	Sig.
	B	SE	β		
IPV (HITS)	0.262	0.051	0.133	5.146	0.000
Irritability (IRQ)	0.230	0.029	0.298	8.061	0.000
Youth Conduct Problem (YCPS)	0.139	0.048	0.076	2.895	0.004
Depression (CES-DC)	0.336	0.025	0.485	13.396	0.000

YCPS-R, Youth Conduct Problems Scale-Rwanda

CES-DC, The Center for Epidemiological Studies Depression Scale for Children

IPV, HITS, Hurt, Insult, Threaten, Screaming

IRQ, The Adapted Irritability Questionnaire

Rsquare = 0.7304

The model consisting of parental intimate partner violence (IPV), depression, anxiety, and youth conduct problem symptoms explained 67% of the total variance of irritability symptoms. The results revealed that parental IPV (β = 0.070, *p* < 0.015), depression (β = 0.398, *p* < 0.001), anxiety (β = 0.361, *p* < 0.001), and youth conduct problems (β = 0.109, *p* < 0.001) were all significantly associated with irritability symptoms among children sampled (Table 6).

**Table 6:** The association between parental intimate partner violence and child’s Irritability symptoms

Variables	Unstandardized coefficients		Standardized coefficients	t	Sig.
	B	SE	β		
IPV (HITS)	0.180	0.074	0.070	2.440	0.015
Youth Conduct Problem (YCPS)	0.260	0.068	0.109	3.808	0.000
Depression (CES-DC)	0.358	0.038	0.398	9.319	0.000
Anxiety (YSR)	0.468	0.058	0.361	8.061	0.000

YCPS-R, Youth Conduct Problems Scale-Rwanda

YSR, Adapted Youth Self-Report -Internalizing Subscale

CES-DC, The Center for Epidemiological Studies Depression Scale for Children

IPV, HITS, Hurt, Insult, Threaten, Screaming

YSR, Adapted Youth Self-Report -Internalizing Subscale

Square= 0.674

On the other hand, the model comprising intimate partner violence, anxiety, irritability, and youth conduct problem symptoms explained 71% of the total variance of depression symptoms. Only anxiety (β = 0.515, *p* < 0.001) and irritability (β = 0.349, *p* < 0.001) were found to be significantly associated with child depression. IPV was not significantly associated with child depression (Table 7).

**Table 7:** The association between parental intimate partner violence and child’s depression Symptoms

Variables	Unstandardized coefficients		Standardized coefficients	t	Sig.
	B	Std. error	β		
IPV	0.037	0.077	0.013	0.472	0.637
Anxiety (YSR)	0.743	0.055	0.515	13.396	0.000
Irritability (IRQ)	0.389	0.042	0.349	9.319	0.000
Youth Conduct Problem (YCPS)	0.108	0.072	0.041	1.501	0.134

YCPS-R, Youth Conduct Problems Scale-Rwanda

YSR, Adapted Youth Self-Report -Internalizing Subscale

IPV, HITS, Hurt, Insult, Threaten, Screaming

YSR, Adapted Youth Self-Report -Internalizing Subscale

Rsquare= 0.713

The model comprised of parental intimate partner violence (IPV) and other symptoms of child mental health outcomes (depression, anxiety, and irritability) explained 71% of the total variance in youth conduct problems. IPV and depression were not found to be significantly associated with youth conduct problems. However, child’s anxiety (β = 0.203, *p* < 0.004) and irritability (β = 0.241, *p* < 0.001) were both significantly associated with youth conduct problems (Table 8).

**Table 8:** The association between parental intimate partner violence and youth Conduct problem

Variables	Unstandardized coefficients		Standardized coefficients	t	Sig.
	B	Std. error	β		
IPV	0.034	0.046	0.032	0.738	0.461
Depression (CES-DC)	0.039	0.026	0.103	1.501	0.134
Anxiety (YSR)	0.110	0.038	0.203	2.895	0.004
Irritability (IRQ)	0.101	0.027	0.241	3.808	0.000

YSR, Adapted Youth Self-Report -Internalizing Subscale

CES-DC, The Center for Epidemiological Studies Depression Scale for Children

IPV, HITS, Hurt, Insult, Threaten, Screaming

YSR, Adapted Youth Self-Report -Internalizing Subscale

Rsquare= 0.713

## Discussion

To our knowledge this is the first study to investigate the relationship of intimate partner violence with child mental difficulties in Rwanda, and one of the handful studies of this relationship in Africa. Among mental difficulties studied, the study found that children were struggling most from irritability; 40.1% from parents’ report and 33.88% from Children’ report. Considering the evidence that irritability is common in children and adolescents [31], being exposed to perpetual parental IPV must be hard on children who are more likely to be annoyed easily, develop emotion of anger, and temper outbursts. According to parents’ account alcohol and drug abuse, poverty, and communication difficulties were the leading factors for IPV. In other words, in addition to parental IPV most of the children were exposed to hardship, and most likely abused and neglected by their parents. In fact, in a separate analysis, IPV as measured by HITS was associated with negative parenting style largely characterized by abuse and neglect.

The study also found considerable prevalence of depression among children. The rates were 26.25% and 23.3% according to children and parents report, respectively. Depression is a public health concern in Rwanda. Over the last decade, depressive disorders have been the third leading cause of years lived with disability (Intitute for Health Metrics and Evalution, 2019). Recent studies of depression among young populations in Rwanda have found higher prevalence of depression in the youth. A study by Schaal and colleagues found a prevalence of 33.8% among orphans of the Genocide [32] while Boris et al. had found a prevalence of 53% among youth-headed households [33].These studies were particularly interested in populations at higher risk, notably survivors of the Genocide who faced adverse events including losing their loved ones, marginalization, high level of grief, and poverty; which could explain higher levels of depression. The risk for developing depression might be lower for children exposed to IPV compared with those surviving life-threatening events such as the Genocide, however, evidence suggest that children exposed to IPV have higher risk of developing internalizing problems such as depression compared with non-exposed [9, 11, 34]. In the Rwandan context, further studies are needed to understand the mechanisms and pathways of the relationship between children exposure to parental IPV and mental difficulties.

Strong and positive correlations between scores on the HITS scale and the scores on the different scales used for the measurement of mental difficulties suggest that the severity of the violence in the families is associated with severe mental difficulties for children living in those families. The scale used for measuring the exposure to IPV during the present study captures the complexity of the violence in its various aspects including physical, emotional and psychological, which provides good insights into the social environment to which children were exposed. It is worthwhile to note that in addition to children witnessing of parental IPV, children are also likely to experience direct violence such as abuse from their parents. The study in Uganda whose social make up is similar to Rwanda found that the overlap between witnessing IPV and experiencing violence for children was almost complete [11]. The present study also found association between parental exposure to IPV and harsh parenting behaviors.

Further, there were also strong relationships between multiple mental difficulties among the children. For instance, the correlation coefficient of 0. 774 was found between CES-DC scores and IRQ scores. CES-DC instrument was used to measure depression while IRQ instrument was used to measure irritability among the children. Such strong relationship found between depression and irritability is supported by the literature. Evidence suggests that irritability is one of the main moods humans experience and it is one of the criteria for depression in the children in DSM-IV [31]. Besides, irritability is one of the criteria for anxiety disorders or one of its characteristics. The present study also found strong correlation between irritability and anxiety disorder among children. In fact, a correlation coefficient of 0.774 was found between IRQ scores and YSR scores. YSR scale was used to measure anxiety among the children. Further, there were strong correlation between depression and anxiety among the children. Depression and anxiety are well known comorbid conditions and the present study found a correlation coefficient of 0.81 between scores on CES-DC and YSR scales. This implies that most of children who showed depression symptoms had also met the criteria for anxiety. Finally, conduct problems among the children were also positively correlated to all mental conditions. Conduct problems are common among children exposed to IPV and could be explained by negative parenting style which is also associated with parental IPV. A randomized clinical trial which trained them on child management skills led to reductions in negative parenting behaviors which, in turn, had significant effects on child conduct problems.

The present study was able to capture the perception of both parents and children about children’ mental health. The similarity in the opinions between these groups is quite remarkable. Except for conduct problems where a significant mean score difference (*p* < 0.05) was observed between children and parents, for other mental difficulties, notably depression, anxiety, and irritability; the mean score differences were not statistically significant (*p* > 0.05). The tendency of agreement of opinions between parents and children suggest that the two groups not only are aware of the effect of the parental IPV on mental health of the children, but also the extent of the problem is broadly shared between these two groups. In fact, comparisons of the prevalence figures for mental difficulties reported during this study reinforce the shared perceptions between parents and children. The stark differences were observed about irritability where children version of the events reported more people compared to parents ‘stories.

Our study has strengths and limitations. One of the strengths is the fact that it has shown that parental intimate partner violence is associated with mental difficulties in their offspring. In the country where the prevalence of domestic violence is high it is very critical to understand its impact on health outcomes including mental health outcomes of the children. The study was also able to provide perception of both the parents and their offspring vis-à-vis mental health outcomes of the latter. There were also limitations to the study. Potential limitations of our study include the following: firstly, this study did not consider different moderating or mediating variables, such as age, gender, parenting styles, maternal mental health, parenting stress, emotional intelligence, and family functioning. Future research should incorporate these variables [35]. Secondly, variables that may influence the reporting of mental health issues, including the duration of exposure to Intimate Partner Violence (IPV), were not accounted for. Research suggests that reports of IPV may only come to light after the issue has persisted for some time [16]. Additionally, the sociocultural understanding of mental health within the community can impact reporting behaviors. In some cultures, there may be a lack of emphasis on mental health concerns, with priority given to meeting basic needs [36]. Consequently, mental health issues among children or parents may not receive adequate attention or may be overlooked. Youth conduct problems may serve as manifestations of underlying issues such as IPV. However, these behaviors may be misinterpreted or categorized as delinquency, leading to a failure to recognize the root cause [37]. Therefore, variables such as the timing of IPV disclosure, sociocultural attitudes towards mental health, and the interpretation of youth conduct behaviors are essential considerations when examining the impact of IPV on mental health outcomes. Additionally, due to cultural beliefs and the sensitive nature of both mental disorders and intimate partner violence (IPV), there is a possibility that some events may have been underreported. Moreover, the participants in the study were selected from among the beneficiaries of a non-governmental organization, thus the results cannot be generalized to the entire population.

## Conclusion

The study revealed that the prevalence of parental IPV has adverse effects on mental health outcomes of their offspring. Also, not only there were associations between IPV and poor mental health outcomes but also interconnections between different mental conditions suggest that children exposed to IPV are more likely to suffer from one mental condition. In country where the prevalence of domestic violence is high, further studies are needed to understand the pathways through which exposure to parental IPV leads to poor mental health of their offspring to be able to design effective interventions for addressing mental health problems among the children.

## Data Availability

The authors declare that no data are available for this article.

## Abbreviations

CES-DC: Center for Epidemiological Studies Depression Scale for Children
DV: Domestic violence
HIC: High Income Countries
HITS: Hurt, Insult, Threaten, Screaming
IPV: Intimate partner violence
IRQ: Adapted Irritability Questionnaire
LIWOHA: Life Wounds Healing Association
PTSD: Posttraumatic Stress Disorders
RDHS: Rwanda Demographic Health Surveys
SDQ: Strengths and Difficulties Questionnaire
WHO: World Health Organisation
YCPS-R: Youth Conduct Problems Scale-Rwanda
YSR: The Adapted Youth Self-Report -Internalizing Subscale

## References

[1] Kusuma Y, Babu B (2017). Elimination of violence against women and girls as a global action agenda. J Inj Violence Res.

[2] Breiding JMBCKSSBMBMMR. Intimate Partner Violence Surveillance: Uniform Defintions and Recommended Data Elements, Atlanta, Georgia, 2015.

[3] Bonomi AE, Anderson ML, Rivara FP, Thompson RS (2007). Health outcomes in women with physical and sexual intimate partner violence exposure. J Womens Health.

[4] Saddki N, Suhaimi AA, Daud R. Maxillofacial injuries associated with intimate partner violence in women. BMC Public Health. 2010;10. 10.1186/1471-2458-10-268. 10.1186/1471-2458-10-268 PMC2882351 20492720

[5] Campbell JC (2002). Health consequences of intimate partner violence. Lancet.

[6] J. M. Golding1, Intimate Partner Violence as a Risk Factor for Mental Disorders: A Meta-Analysis, 1999.

[7] Chander P, et al. Intimate partner violence and child behavioral problems in South Africa. Pediatrics. 2017;139(3). 10.1542/peds.2016-1059. 10.1542/peds.2016-1059 PMC5330393 28242862

[8] Ferrari G, et al. Domestic violence and mental health: a cross-sectional survey of women seeking help from domestic violence support services. Glob Health Action. 2016;9(1). 10.3402/gha.v9.29890. 10.3402/gha.v9.29890 PMC4748088 26860876

[9] Moylan CA, Herrenkohl TI, Sousa C, Tajima EA, Herrenkohl RC, Russo MJ (2010). The effects of child abuse and exposure to domestic violence on adolescent internalizing and externalizing behavior problems. J Fam Violence.

[10] Sonego M, Pichiule M, Gandarillas A, Polo C, Ordobás M (2018). Mental health in girls and boys exposed to intimate partner violence. Public Health.

[11] Devries KM et al. Witnessing intimate partner violence and child maltreatment in Ugandan children: A cross-sectional survey, BMJ Open, vol. 7, no. 2, pp. 1–9, 2017, 10.1136/bmjopen-2016-013583. 10.1136/bmjopen-2016-013583 PMC5337724 28246136

[12] Rurangirwa AA, Mogren I, Ntaganira J, Govender K, Krantz G. Intimate partner violence during pregnancy in relation to non-psychotic mental health disorders in Rwanda : a cross-sectional population-based study, pp. 1–9, 2018, 10.1136/bmjopen-2018-021807. 10.1136/bmjopen-2018-021807 PMC6082444 29997142

[13] Rurangirwa AA, Mogren I, Ntaganira J, Krantz G. Intimate partner violence among pregnant women in Rwanda, its associated risk factors and relationship to ANC services attendance: a population-based study. BMJ Open. 2017;7(2). 10.1136/bmjopen-2016-013155. 10.1136/bmjopen-2016-013155 PMC5337709 28399509

[14] Laanpere M, Ringmets I, Part K, Karro H (2013). Intimate partner violence and sexual health outcomes: a population-based study among 16-44-year-old women in Estonia. Eur J Public Health.

[15] Stöckl H (2013). The global prevalence of intimate partner homicide: a systematic review. Lancet.

[16] Bahati C, Izabayo J, Munezero P, Niyonsenga J, Mutesa L. Trends and correlates of intimate partner violence (IPV) victimization in Rwanda: results from the 2015 and 2020 Rwanda Demographic Health Survey (RDHS 2015 and 2020), BMC Womens Health, vol. 22, no. 1, p. 368, 2022, 10.1186/s12905-022-01951-3. 10.1186/s12905-022-01951-3 PMC9447352 36068627

[17] Rurangirwa AA, Mogren I, Ntaganira J, Govender K, Krantz G (2018). Intimate partner violence during pregnancy in relation to non-psychotic mental health disorders in Rwanda: a cross-sectional population-based study. BMJ Open.

[18] Bahati C, Rukundo G, Nyirahabimana N, Izabayo J, Niyonsenga J, Sezibera V, February. 114465, 2022, doi: 10.1016/j.psychres.2022.114465. 10.1016/j.psychres.2022.114465 35219265

[19] Mannell J, Jackson S, Umutoni A. Women’s responses to intimate partner violence in Rwanda: rethinking agency in constrained social contexts. Glob Public Health. Feb. 2016;11:1–2. 10.1080/17441692.2015.1013050. 10.1080/17441692.2015.1013050 25734771

[20] Pun KD, et al. Community perceptions on domestic violence against pregnant women in Nepal: a qualitative study. Glob Health Action. 2016;9(1). 10.3402/GHA.V9.31964. 10.3402/gha.v9.31964 PMC5122230 27882865

[21] Evans SE, Davies C, DiLillo D. Exposure to domestic violence: a meta-analysis of child and adolescent outcomes. Aggress Violent Beh. Mar. 2008;13:131–40. 10.1016/j.avb.2008.02.005. no. 2.

[22] Verduin F, Engelhard EAN, Rutayisire T, Stronks K, Scholte WF (2013). Intimate Partner violence in Rwanda: the Mental Health of victims and perpetrators. J Interpers Violence.

[23] Umubyeyi A, Mogren I, Ntaganira J, Krantz G. Intimate partner violence and its contribution to mental disorders in men and women in the post genocide Rwanda: findings from a population based study. BMC Psychiatry. 2014;14(1). 10.1186/s12888-014-0315-7. 10.1186/s12888-014-0315-7 PMC4245842 25406929

[24] Howell KH, Cater ÅK, Miller-Graff LE, Graham-Bermann SA. The Process of Reporting and Receiving Support Following Exposure to Intimate Partner Violence During Childhood, J Interpers Violence, vol. 30, no. 16, pp. 2886–2907, Oct. 2015, 10.1177/0886260514554289. 10.1177/0886260514554289 25389193

[25] Bair-Merritt MH, Crowne SS, Thompson DA, Sibinga E, Trent M, Campbell J (2010). Why do women use intimate Partner violence? A systematic review of women’s motivations. Trauma Violence Abuse.

[26] Betancourt TS et al. Family-based prevention of mental health problems in children affected by HIV and AIDS: An open trial, Aids, vol. 28, no. SUPPL. 3, 2014, 10.1097/QAD.0000000000000336. 10.1097/QAD.0000000000000336 PMC4315615 24991909

[27] Rabin RF, Jennings JM, Campbell JC, Bair-Merritt MH (2009). Intimate Partner Violence Screening Tools. A systematic review. Am J Prev Med.

[28] Ng LC, Kanyanganzi F, Munyanah M, Mushashi C, Betancourt TS. Developing and validating the Youth Conduct problems Scale-Rwanda: a mixed methods approach. PLoS ONE. 2014;9(6). 10.1371/journal.pone.0100549. 10.1371/journal.pone.0100549 PMC4065113 24949628

[29] Betancourt TS (2017). Family-based promotion of mental health in children affected by HIV: a pilot randomized controlled trial. J Child Psychol Psychiatry.

[30] Craig KJ, Hietanen H, Markova IS, Berrios GE (2008). The Irritability Questionnaire: a new scale for the measurement of irritability. Psychiatry Res.

[31] Stringaris A (2011). Irritability in children and adolescents: a challenge for DSM-5. Eur Child Adolesc Psychiatry.

[32] Schaal S, Dusingizemungu J-P, Jacob N, Elbert T (2011). Rates of trauma spectrum disorders and risks of posttraumatic stress disorder in a sample of orphaned and widowed genocide survivors. Eur J Psychotraumatol.

[33] Boris NW et al. Depressive symptoms in Youth heads of Household in Rwanda, 162, 9, pp. 836–43, 2008. 10.1001/archpedi.162.9.836 18762600

[34] Holt S, Buckley H, Whelan S (2008). The impact of exposure to domestic violence on children and young people: a review of the literature. Child Abuse Negl.

[35] Carter B, Paranjothy S, Davies A, Kemp A. Mediators and Effect Modifiers of the Causal Pathway Between Child Exposure to Domestic Violence and Internalizing Behaviors Among Children and Adolescents: A Systematic Literature Review, Trauma, Violence, and Abuse, vol. 23, no. 2. SAGE Publications Ltd, pp. 594–604, Apr. 01, 2022. 10.1177/1524838020965964. 10.1177/1524838020965964 PMC8905123 33094689

[36] Ngui EM, Khasakhala L, Ndetei D, Roberts LW. Mental disorders, health inequalities and ethics: A global perspective, International Review of Psychiatry, vol. 22, no. 3. pp. 235–244, Jun. 2010. 10.3109/09540261.2010.485273. 10.3109/09540261.2010.485273 PMC2935265 20528652

[37] Li SD, Xiong R, Liang M, Zhang X, Tang W. Pathways From Family Violence to Adolescent Violence: Examining the Mediating Mechanisms, Front Psychol, vol. 12, Feb. 2021, 10.3389/fpsyg.2021.611006. 10.3389/fpsyg.2021.611006 PMC7900619 33633642

